# A longitudinal study of gene expression in first-episode schizophrenia; exploring relapse mechanisms by co-expression analysis in peripheral blood

**DOI:** 10.1038/s41398-021-01645-8

**Published:** 2021-10-19

**Authors:** P. Gassó, N. Rodríguez, A. Martínez-Pinteño, G. Mezquida, M. Ribeiro, J. González-Peñas, I. Zorrilla, L. Martínez-Sadurni, R. Rodriguez-Jimenez, I. Corripio, S. Sarró, A. Ibáñez, J. Usall, A. Lobo, C. Moren, M. J. Cuesta, M. Parellada, A. González-Pinto, E. Berrocoso, M. Bernardo, S. Mas, 2EPs Group, M. Bioque, S. Amoretti, A. Andreu-Bernabeu, X. Gurriarán, A. Alonso-Solís, E. Grasa, P. López, E. Garcia, D. Bergé, A. Trabsa, L. Sànchez-Pastor, O. Jiménez-Rodríguez, E. Pomarol-Clotet, I. Feria-Raposo, A. Butjosa, M. Pardo, L. Moreno-Izco, A. M. Sánchez-Torres, J. Saiz-Ruiz, L. León-Quismondo, J. Nacher, F. Contreras, C. De-la-Cámara, M. Gutiérrez, P. A. Sáiz

**Affiliations:** 1 Department of Clinical Foundations, Pharmacology Unit, University of Barcelona, Barcelona, Spain; 2 Institut d’investigacions Biomèdiques August Pi i Sunyer (IDIBAPs), Barcelona, Spain; 3 Barcelona Clínic Schizophrenia Unit (BCSU), Neuroscience Institute, Hospital Clínic de Barcelona, Barcelona, Spain; 4 Centro de Investigación Biomédica en Red en Salud Mental (CIBERSAM), Madrid, Spain; 5 Department of Psychiatry, Complejo Hospitalario de Navarra, Pamplona, Spain; 6 IdiSNA, Navarra Institute for Health Research, Pamplona, Spain; 7 Department of Child and Adolescent Psychiatry, Institute of Psychiatry and Mental Health, Hospital General Universitario Gregorio Marañón, IiSGM, School of Medicine, Universidad Complutense, Madrid, Spain; 8 Department of Psychiatry, Hospital Universitario de Alava, Vitoria, Spain; 9 BIOARABA Health Research Institute, Vitoria, Spain; 10 University of the Basque Country, Vitoria, Spain; 11 Hospital del Mar Medicar Research Institute (IMIM), Barcelona, Spain; 12 Instituto de Investigación Sanitaria Hospital 12 de Octubre (imas12), Madrid, Spain; 13 CogPsy Group, Universidad Complutense de Madrid (UCM), Madrid, Spain; 14 Psychiatry Department, Institut d’Investigació Biomèdica-Sant Pau (IIB-SANT PAU), Hospital de la Santa Creu i Sant Pau, Barcelona, Spain; 15 Universitat Autònoma de Barcelona (UAB), Barcelona, Spain; 16 FIDMAG Germanes Hospitalàries Research Foundation, Barcelona, Spain; 17 School of Medicine, Universitat Internacional de Catalunya, Barcelona, Spain; 18 Department of Psychiatry, Hospital Universitario Ramón y Cajal, IRYCIS, Universidad de Alcalá, Madrid, Spain; 19 Etiopatogènia i tractament dels trastorns mentals greus (MERITT) Institut de Recerca Sant Joan de Déu Parc Sanitari Sant Joan de Déu, Barcelona, Spain; 20 Department of Medicine and Psychiatry, Universidad de Zaragoza, Zaragoza, Spain; 21 Instituto de Investigación Sanitaria Aragón (IIS Aragón), Zaragoza, Spain; 22 Cellex, IDIBAPS, University of Barcelona-Hospital Clínic of Barcelona, Barcelona, 08036 Spain; 23 Centro de Investigación Biomédica en Red (CIBER) de Enfermedades Raras (CIBERER), Madrid, 28029 Spain; 24 Neuropsychopharmacology and Psychobiology Research Group, Department of Psychology, University of Cádiz, Cádiz, Spain; 25 Instituto de Investigación e Innovación Biomédica de Cádiz, INiBICA, Hospital Universitario Puerta del Mar, Cádiz, Spain; 26 Department of Medicine, University of Barcelona, Barcelona, Spain; 27 Hospital del Mar Medical Research Institute (IMIM), Barcelona, Spain; 28 Autonomous University of Barcelona, Barcelona, Spain; 29 Benito Menni CASM, Sant Boi de Llobregat, Spain; 30 Institut de Recerca Sant Joan de Déu, Hospital Sant Joan de Déu, Esplugues de Llobregat, Spain; 31 Department of Psychiatry, Hospital Universitario Ramón y Cajal, Madrid, Spain; 32 Neurobiology Unit, Program in Neurosciences and Institute of Biotechnology and Biomedicine (BIOTECMED), Universitat de València, Burjassot, Spain; 33 Biomedical Research Networking Centre in Mental Health (CIBERSAM), Madrid, Spain; 34 Biomedical Research Institute INCLIVA, Valencia, Spain; 35 Bellvitge Biomedical Research Institute IDIBELL, Department of Psychiatry, Bellvitge University Hospital, Hospitalet de Llobregat, Barcelona, Spain; 36 Biomedical Research Networking Center for Mental Health Network (CIBERSAM), Barcelona, Spain; 37 Hospital Clínico Universitario and Instituto de Investigación Sanitaria (IIS) Aragón, Zaragoza, Spain; 38 Araba University Hospital, Bioaraba Research Institute, Psychiatry, Vitoria, Spain; 39 Department of Neurosciences, University of the Basque Country (UPV/EHU), Leioa, Spain; 40 Department of Psychiatry, School of Medicine, University of Oviedo, Instituto de Investigación Sanitaria del Principado de Asturias, Mental Health Services of Principado de Asturias, Biomedical Research Networking Centre in Mental Health (CIBERSAM), Oviedo, Spain

**Keywords:** Personalized medicine, Prognostic markers

## Abstract

Little is known about the pathophysiological mechanisms of relapse in first-episode schizophrenia, which limits the study of potential biomarkers. To explore relapse mechanisms and identify potential biomarkers for relapse prediction, we analyzed gene expression in peripheral blood in a cohort of first-episode schizophrenia patients with less than 5 years of evolution who had been evaluated over a 3-year follow-up period. A total of 91 participants of the 2EPs project formed the sample for baseline gene expression analysis. Of these, 67 provided biological samples at follow-up (36 after 3 years and 31 at relapse). Gene expression was assessed using the Clariom S Human Array. Weighted gene co-expression network analysis was applied to identify modules of co-expressed genes and to analyze their preservation after 3 years of follow-up or at relapse. Among the 25 modules identified, one module was semi-conserved at relapse (DarkTurquoise) and was enriched with risk genes for schizophrenia, showing a dysregulation of the *TCF4* gene network in the module. Two modules were semi-conserved both at relapse and after 3 years of follow-up (DarkRed and DarkGrey) and were found to be biologically associated with protein modification and protein location processes. Higher expression of DarkRed genes was associated with higher risk of suffering a relapse and early appearance of relapse (*p* = 0.045). Our findings suggest that a dysregulation of the *TCF4* network could be an important step in the biological process that leads to relapse and suggest that genes related to the ubiquitin proteosome system could be potential biomarkers of relapse.

## Introduction

Despite the efficacy of antipsychotic medication in the treatment of schizophrenia-spectrum disorders, this condition is still characterized by persistent functional impairment and recurrent psychotic relapses for most patients. Relapse is characterized by acute psychotic exacerbation and is considered a biologically toxic event that leads to progressive illness course and an overall deterioration of psychosocial functioning [1, 2]. This, in turn, has been associated with progressive loss of cortical tissue and overall brain volume [3]. The frequency and the cumulative effects of relapse are related to poor long-term outcomes, a decline in quality of life and lower response to subsequent treatment, and it also often requires inpatient hospitalization, increasing healthcare costs [4].

Around 41–63% of patients will experience a relapse within the first 3 years of a first episode of psychosis (FEP) [5] and up to 80% within the first 5 years of remission from a FEP [6]. The main risk factor for relapse is non-adherence to antipsychotic medication [7, 8]. However, 20–30% of individuals complying with treatment will develop subsequent relapses [9, 10].

Considering that relapse represents much of the personal and social burden of schizophrenia, there is a need to develop more efficient interventions or treatment recommendations to prevent relapse. In this scenario, the search for the potential role of biomarkers to stratify patients by their individual characteristics according to their risk of relapse is gaining attention. It has been proposed that stratification could be undertaken after clinical remission with antipsychotics [11].

Nevertheless, the lack of understanding about the pathophysiological mechanisms of relapse hinders research into potential biomarkers and the development of preventive and therapeutic interventions. Although different mechanisms have been proposed, it appears that dopamine represents the final common pathway in relapse [12]. This agrees with the proposed dopamine super sensitivity theory, wherein the chronic blockade of dopamine receptors results in compensatory receptor upregulation, which in turn leads to an increased sensitivity to endogenous dopamine that leaves individuals more prone to relapse upon antipsychotic discontinuation [13, 14]. The worsening of long-term outcomes after chronic exposure to antipsychotics is generating again an intense debate [15, 16]. Several moderators that influence neurodevelopment, such as stress [17], may impact this process, and several potentially state-dependent biomarkers have been proposed such as inflammatory markers [18, 19] or neurotrophins [20**–**22].

To uncover relapse mechanisms, we investigated a molecular phenotype such as gene expression as an intermediate measure between genetic and clinical variation. To this end, we analyzed the preservation of blood co-expression modules (clusters of genes with highly correlated expression) in a cohort of first-episode schizophrenia patients with less than 5 years of evolution evaluated over a 3-year follow-up period. Co-expression modules were defined at study enrollment with patients under remission of their symptoms. Preservation of these modules was assessed at relapse or after 3 years of enrollment for those patients who had not experienced relapse. Non-preserved or semi-conserved modules at relapse are expected to be of relevance in its pathophysiology and are potential biomarkers of relapse.

## Material and methods

This study is part of the project “Clinical and neurobiological determinants of second episodes of schizophrenia. Longitudinal study of first episode of psychosis” (PI11/00325) (2EPs Project), which arose to identify and characterize those clinical, environmental and biological factors that predict a relapse.

### Study design

The 2EPs is a naturalistic, multicenter, coordinated, and multimodal study of patients with a first psychotic episode of schizophrenia with less than 5 years of evolution, and takes a 3-year longitudinal-prospective follow-up design. The project includes six modules: general, neuroimaging, adherence, neurocognition, physical health, and biological. Due to its main goals, the present study was framed within the general and biological modules. The first one assesses the presence or absence of relapses and includes the clinical assessments. The biological module searches for biomarkers potentially involved in second episodes [23].

### Subjects

The inclusion criteria from the 2EPs Project were: (a) age between 16 and 40 years at the time of first assessment (baseline visit); (b) meeting diagnostic criteria according to DSM-IV for schizophrenia or schizophreniform disorder [24]; (c) being in remission according to Andreassen’s criteria [25] from the first psychotic episode (which should have occurred within the last 5 years); (d) not having relapsed after the first psychotic episode; (e) speaking Spanish correctly; and (f) providing the signed informed consent form. The exclusion criteria were: (a) having experienced a traumatic brain injury with loss of consciousness; (b) presenting mental retardation understood not only as IQ < 70, but also presenting malfunctioning and problems with adaptive process, and/or (c) presenting somatic pathology with mental repercussion.

From the initial 223 patients recruited in the 2EPs Project, 91 (40.8%) participated in the biological module and provided a biological sample for gene expression analysis at baseline. Of these, 67 (73.6%) provided biological samples at follow-up (36 after 3 years and 31 at relapse).

The study was approved by the investigation ethics committees of all participating clinical centers. Informed consent was obtained from all participants. For children under the age of 18 years old, parents or legal guardians gave written informed consent before the beginning of their participation in the study, and patients assented to participate. When requested, participants in the study were given a report on the results of the tests. This study was conducted in accordance with the Declaration of Helsinki.

### Clinical assessment

Demographic data were collected for all patients through semi-structured interviews. Diagnoses were determined according to the DSM-IV criteria (American Psychiatric Association, 1994), with the SCID-I [26] or the Kiddie-SADS [27] depending on age.

Clinical symptomatology was assessed using the Spanish validated version of the Positive and Negative Syndrome Scale (PANSS) [28]. Pharmacological treatment was also recorded during all the visits. The extent of medication non-adherence was assessed using the Morisky Green Levine Medication Adherence Scale [29].

### Sample collection, RNA isolation, and microarray hybridization

Ten milliliter of peripheral blood was collected at baseline and at follow-up (3 years or at relapse) in PAXgene Blood RNA tubes (PreAnalytiX Gmbh, Switzerland). Total RNA was isolated in accordance with the manufacturer’s protocol (PAXgene Blood RNA kit, PreAnalytiX Gmbh, Switzerland). The purity and integrity of RNA was assessed using an Agilent 2100 Bioanalyzer (Agilent Technologies, Palo Alto, CA, USA). One microgram of purified RNA from each of sample was submitted to the Kompetenzzentrum für Fluoreszente Bioanalytik Microarray Technology (KFB, BioPark Regensburg GmbH, Regensburg, Germany) for labeling and hybridization to Clariom S Human Array (Affymetrix, Santa Clara, CA, USA), following the manufacturer’s protocols. The Clariom S Human Array comprises more than 221,300 probes covering over 337,100 transcripts and variants, which in turn represent 20,800 genes.

### Genome-wide expression analysis and the WGCNA procedure

Microarray data preprocessing was performed using the Oligo R package [30]. The data were standardized using robust multichip analysis. Multiple probes mapping to the same gene were merged using the average as the summary of the hybridization values. Co-expression modules were identified using the R software package for weighted gene co-expression network analysis (WGCNA) [31]. Firstly, in order to remove outlier samples, distance-based adjacency matrices of samples were estimated and sample network connectivity according to the distances was standardized. Samples with connectivity less than −5 were considered as outliers and were excluded (Supplementary Fig. 1A). Based on the assumption that non- or semi-preserved modules between remission and relapse may be functionally related to relapse mechanisms, baseline samples were considered as the reference set for module detection. The co-expression analysis involved constructing a matrix of pairwise correlations between all pairs of genes across all selected samples. Next, the matrix was raised to a soft-thresholding power (*β* = 6 in this study) to obtain an adjacency matrix (Supplementary Fig. 1B). To identify modules of co-expressed genes, we constructed the topological overlap-based dissimilarity, which was then used as input to average linkage hierarchical clustering. This step resulted in a clustering tree (dendrogram) whose branches were identified for cutting based on their shape, using the dynamic tree-cutting algorithm (Supplementary Fig. 1C). The above steps were performed using the automatic network construction and module detection function (blockwiseModules in WGCNA), with the following parameters: minModuleSize of 30, reassignThreshold of 0, and mergeCutHeight of 0.25.

### Preservation analysis

Module preservation analysis allowed us to evaluate how well the modular structure of the baseline samples is preserved after 36 months or at relapse. To do this, according to Langfelder et al. [32], the function modulePreservation of WGCNA package was used and a permutation test (based on the generation of 200 random permutations) was calculated, which assesses the preservation of the connectivity and density of each network module. We used composite module preservation statistics that are constructed to summarize changes in module preservation [32]. Zsummary is a composite module preservation statistic that simultaneously assesses whether the genes in a defined module in the baseline samples remain highly connected after 36 months (or at relapse) and investigates whether the connectivity patterns between the genes in the baseline samples remain similar, compared with the connectivity after 36 months or relapse samples. The modules with Zsummary > 10 were interpreted as highly preserved, if 2 < Zsummary < 10 were defined as semi-conserved and the modules with Zsummary < 2 were considered to be non-preserved following Langfelder et al. [32]. The main advantage of this score is that it allowed threshold definition, although it often depends on module size. We also calculated Zdensity, a density-based preservation statistic used to determine whether the genes in a reference module remain highly connected in the test network (Zdensity > 10, density preservation); and Zconnectivity, a preservation statistic that assesses whether the overall connectivity pattern between genes in a reference module is similar in the reference and test networks (Zconnectivity > 5, preserved connectivity) [32]. As a complementary statistic we also computed medianRank, a composite score based on the ranks of the observed preservation statistics, which shows no dependence on the module size. The main disadvantage of medianRank is that it is only applicable for ranking modules, given relative preservation information of the modules.

Network visualization of selected modules showing the correlation network adjacencies was performed using Cytoscape 3.8.2 software.

### Characterization of relevant modules

To characterize non-preserved or semi-conserved modules, two approaches were followed: (1) gene set enrichment analysis of Gene Ontology terms (Biological Process) and Reactome pathways were assessed using gProfiler R package [33] (“moderate” hierarchical filtering was used and only pathways containing between 10 and 2000 genes and FDR-adjusted *p* < 0.05 were accepted); and (2) the genes in each module were used to define a gene set, and each such gene set was tested for overlap with gene sets formed by:

- differentially expressed genes in dorsolateral prefrontal cortex (DLPFC) between subjects with schizophrenia (*N* = 159) versus control (*n* = 293) subjects [34];
- differentially expressed genes in DLPFC between subjects with schizophrenia (*N* = 258) versus control (*n* = 279) subjects [35];
- genes associated with schizophrenia using gene expression imputation (transcriptome-wide analysis) across multiple brain regions in 40,299 schizophrenia cases and 65,264 matched controls [36];
- genes that have association with common genome-wide association study (GWAS) meta-analysis of the CLOZUK and independent Psychiatric Genomic Consortium datasets, excluding related and overlapping samples (total of 40,675 cases and 64,643 controls) [37].

Gene overlap was assessed using GeneOverlap R package [38].

### Statistical procedures

Differential gene expression of selected genes was assessed using the Limma R package [39]. Module eigengenes (the first principal component of each module computed in the WGCNA) at baseline were used to test the relapse predictive properties of selected modules using nonparametric receiver operating characteristic (ROC) curve analysis, as well as to test the effect of selected modules on the length of time (months from baseline) taken to reach relapse using Kaplan–Meier and Cox regression analysis. ROC and survival analysis were assessed using IBM SPSS Statistics 20.0 (statistical analysis software, IBM, Chicago, IL, USA).

## Results

Table 1 shows the demographic, clinical, and pharmacological characteristics of the study participants at baseline (*N* = 91), at relapse (*N* = 31), and after 3 years of follow-up if they had not experienced a relapse (*N* = 36). Non-significant differences in baseline variables were observed between the three groups of patients. As could be expected, significant differences were observed in clinical symptomatology at follow-up, with patients at relapse exhibiting a significant worsening of their symptoms with respect to baseline and to patients that had not experienced relapse after 3 years of follow-up (post-hoc analysis *p* < 0.001). However, baseline symptomatology did not differ between patients who suffer a relapse and patients who had not experienced relapse (total PANSS 48.5 ± 14.6 vs. 48.5 ± 14.4, *F*_1_ = 0.935, *p* = 0.966). Regarding pharmacological treatment at follow-up, patients who relapsed were treated more frequently with olanzapine (*p* = 0.02), and received anxiolytics (*p* = 0.007) and lithium (*p* = 0.016) more often than patients who had not experienced a relapse.

**Table 1:** Demographic, clinical, and pharmacological characteristics of: the 91 participants who provided biological sample at baseline; the 36 participants who provided biological sample at baseline and after 3-year follow-up; and the 31 participants who provided biological sample at baseline and at relapse.

	Baseline	Follow-up	Relapse	Statistic
*N*	91	36	31	
*Age, mean* *±* *SD*	25.33 ± 5.85	25.47 ± 5.61	24.87 ± 5.40	*F*_2_ = 0.1, *p* = 0.902
*Age at first diagnosis, mean* *±* *SD*	24.10 ± 5.73	23.83 ± 5.43	23.43 ± 5.27	*F*_2_ = 0.0, *p* = 0.968
*Gender, male, n (%)*	62 (67.4)	28 (77.7)	20 (64.5)	*X*^2^_2_ = 1.6, *p* = 0.447
*Ethnicity, Caucasian, N (%)*	81 (88.0)	31 (86.1)	30 (96.7)	*X*^2^_2_ = 2.2, *p* = 0.323
*Symptomatology*				
PANSS positive (mean ± SD)	9.6 ± 3.2	9.8 ± 4.9	20.3 ± 8.1	*F*_2_ = 57.0, *p* < 0.001
PANSS negative (mean ± SD)	13.9 ± 5.4	12.4 ± 5.1	19.3 ± 8.2	*F*_2_ = 12.4, *p* < 0.001
PANSS general (mean ± SD)	25.6 ± 7.9	23.6 ± 8.9	39.3 ± 14.6	*F*_2_ = 27.2, *p* < 0.001
PANSS total (mean ± SD)	49.1 ± 14.7	45.8 ± 17.3	78.8 ± 28.2	*F*_2_ = 34.0, *p* < 0.001
*Antipsychotic*				
Aripiprazol, *N* (%)	34 (40.5)	15 (57.7)	10 (34.5)	*X*^2^_2_ = 3.3, *p* = 0.186
Paliperidone, *N* (%)	24 (28.6)	7 (26.9)	7 (24.1)	*X*^2^_2_ = 0.2, *p* = 0.897
Risperidone, *N* (%)	13 (15.4)	1 (3.8)	5 (17.2)	*X*^2^_2_ = 2.6, *p* = 0.262
Olanzapine, *N* (%)	12 (14.3)	1 (3.8)	9 (31.0)	*X*^2^_2_ = 7.9, *p* = 0.02
Clozapine, *N* (%)	6 (7.1)	6 (23.1)	4 (13.8)	*X*^2^_2_ = 5.1, *p* = 0.07
Quetiapine, *N* (%)	3 (3.6)	0 (0.0)	2 (6.9)	*X*^2^_2_ = 1.8, *p* = 0.390
Amisulpride, *N* (%)	3 (3.6)	0 (0.0)	0 (0.0)	*X*^2^_2_ = 2.0, *p* = 0.366
Etamine, *N* (%)	2 (2.4)	0 (0.0)	0 (0.0)	*X*^2^_2_ = 1.3, *p* = 0.514
Asenapine, *N* (%)	1 (1.2)	0 (0.0)	1 (3.4)	*X*^2^_2_ = 1.2, *p* = 0.537
Haloperidol, *N* (%)	1 (1.2)	0 (0.0)	0 (0.0)	*X*^2^_2_ = 0.6, *p* = 0.719
Ziprasidone, *N* (%)	1 (1.2)	0 (0.0)	1 (3.4)	*X*^2^_2_ = 1.2, *p* = 0.537
*Co-medication*				
Antidepressant, yes, *N* (%)	24 (28.6)	7 (26.9)	4 (13.8)	*X*^2^_2_ = 2.5, *p* = 0.279
Anxiolytic, yes, *N* (%)	13 (15.5)	2 (7.7)	11 (37.9)	*X*^2^_2_ = 9.6, *p* = 0.007
Lithium, yes, *N* (%)	2 (2.4)	0 (0.0)	4 (13.8)	*X*^2^_2_ = 8.2, *p* = 0.016
Antiepileptic, yes, *N* (%)	5 (5.9)	2 (7.7)	1 (3.4)	*X*^2^_2_ = 0.4, *p* = 0.790
Antiparkinsonian, yes, *N* (%)	11 (13.1)	1 (3.8)	3 (10.3)	*X*^2^_2_ = 1.7, *p* = 0.412
*Adherence*				
Morinsky–Green (mean ± SD)	1.3 ± 0.9	1.3 ± 0.7	1.6 ± 1.1	*F*_2_ = 1.6, *p* = 0.259
Non-adherent, *N* (%)	7 (8.5)	3 (11.1)	6 (20.7)	*X*^2^_2_ = 3.1, *p* = 0.218

Taking baseline samples (*N* = 90) as the reference set, we identified 25 modules of co-expressed genes (Supplementary Fig. 1C). The inferred modules showed different sizes from 41 (DarkGrey module) to 5627 genes (Turquoise module). Also, 1901 genes were assigned to the gray module, which represents the genes that were not co-expressed based on gene dissimilarity measures and were not assigned to any of the modules.

**Fig. 1:**
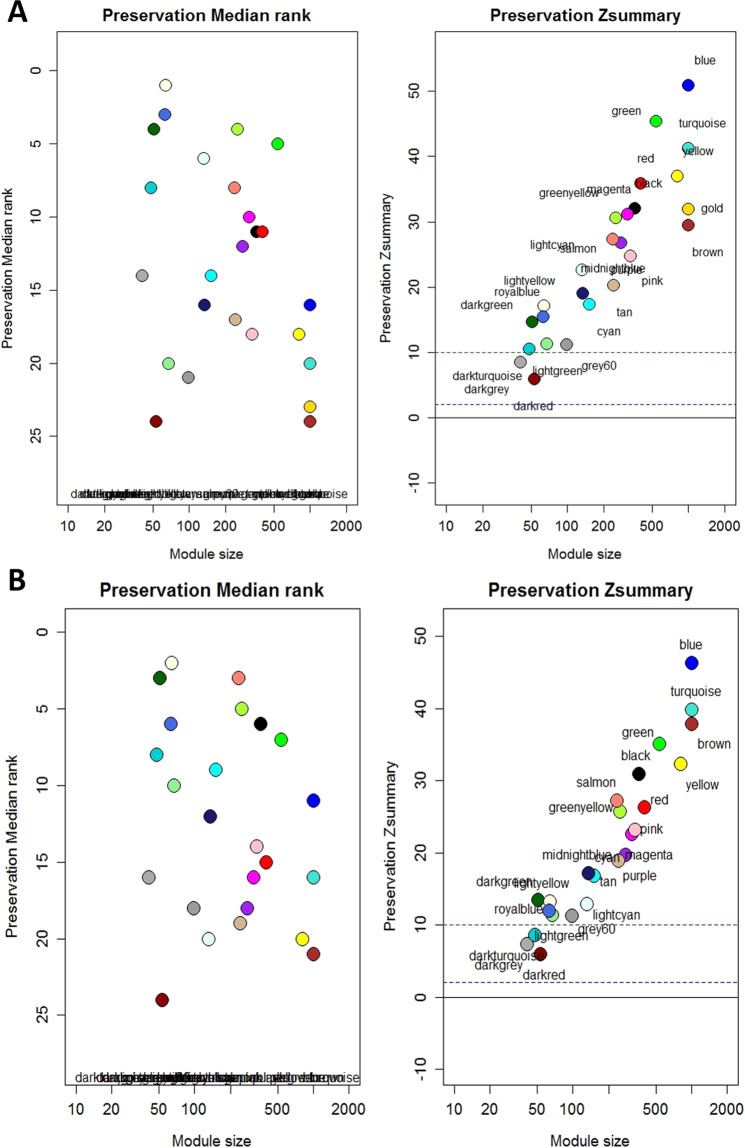
Module preservation analysis. Scatter plot of the meadianRank and Zsummary composite module preservation statistic and module size in **A** samples after 3-year follow-up and **B** samples at relapse. The modules with Zsummary > 10 were interpreted as highly preserved, if 2 < Zsummary < 10 were defined as semi-conserved and the modules with Zsummary < 2 were considered to be non-preserved.

Module preservation analysis, using *z*-score statistics, revealed two semi-conserved modules after 3 years of follow-up: DarkRed (53 genes, Zsummary = 5.9, Zdensity = 4.2, Zconnectivity = 7.4) and DarkGrey (41 genes, Zsummary = 8.6, Zdensity = 9.1, Zconnectivity = 7.9) (Fig. 1A). At relapse, module preservation analysis identified three semi-conserved modules: DarkRed (53 genes, Zsummary = 5.9, Zdensity = 4.3, Zconnectivity = 7.5), DarkGrey (41 genes, Zsummary = 7.4, Zdensity = 6.7, Zconnectivity = 7.9) and DarkTurquoise (48 genes, Zsummary = 8.7, Zdensity = 8.9, Zconnectivity = 7.5) (Fig. 1B). The analysis of the medianRank score confirmed the loss of preservation of the DarkRed and DarkGrey modules in both, non-relapsed (Fig. 1A) and relapsed patients (Fig. 1B). In contrast, results regarding the semi-preservation of the DarkTurquoise module in relapsed patients should be considered with caution. According to the medianRank, the DarkTurquoise module—not showing high preservation compared to other modules with similar size—is far from semi-preserved modules such as DarkRed and DarkGrey, indicating that Zsummary results for DarkTurquoise module could be influenced by module size instead of relapse.

Since the relapsed and non-relapsed samples at follow-up are subsets of the basal sample, differences in module preservation may be related to this different sample composition. As a complementary analysis we performed the preservation analysis using the common samples in the reference and test sets for both, relapsed and non-relapsed sets of patients. This analysis did not shown any difference with the initial analysis (Supplementary Fig. 2).

To investigate the biological functions of the genes included in the semi-conserved modules (Supplementary Table 1), a gene set enrichment analysis was performed. Seventeen biological processes from Gene Ontology and six Reactome pathways were found to be enriched in the DarkRed module, mainly related to detection of stimulus and protein ubiquitination processes. The DarkGrey module was enriched with four Biological processes related to protein location. No enrichment of biological processes and Reactome pathways were detected in the DarkTurquoise module. Table 2 shows the identified significant terms for each module.

**Table 2:** Significant terms (biological processes from Gene Ontology and Reactome pathways) of the gene set enrichment analysis performed in the three semi-conserved modules identified in the present study.

Module	Term_id	Term_name	Adjusted *p* value	Term size	Term list
DarkRed	GO:0050911	Detection of chemical stimulus involved in sensory perception of smell	1 × 10^−9^	396	13
DarkRed	GO:0007600	Sensory perception	8 × 10^−7^	970	14
DarkRed	GO:0007186	G protein-coupled receptor signaling pathway	7 × 10^−6^	1361	15
DarkRed	GO:0016579	Protein deubiquitination	1 × 10^−5^	291	8
DarkRed	GO:0070646	Protein modification by small protein removal	1 × 10^−5^	309	8
DarkRed	GO:0050877	Nervous system process	9 × 10^−5^	1465	14
DarkRed	GO:0003008	System process	1 × 10^−2^	2327	14
DarkRed	GO:0006511	Ubiquitin-dependent protein catabolic process	2 × 10^−2^	657	7
DarkRed	GO:0019941	Modification-dependent protein catabolic process	2 × 10^−2^	664	7
DarkRed	GO:0043632	Modification-dependent macromolecule catabolic process	2 × 10^−2^	675	7
DarkRed	GO:0051603	Proteolysis involved in cellular protein catabolic process	4 × 10^−2^	756	7
DarkRed	REAC:R-HSA-381753	Olfactory Signaling Pathway	1 × 10^−8^	380	11
DarkRed	REAC:R-HSA-418555	G alpha (s) signaling events	1 × 10^−7^	522	11
DarkRed	REAC:R-HSA-5689880	Ub-specific processing proteases	3 × 10^−6^	200	7
DarkRed	REAC:R-HSA-5688426	Deubiquitination	2 × 10^−5^	276	7
DarkRed	REAC:R-HSA-388396	GPCR downstream signaling	9 × 10^−5^	1088	11
DarkRed	REAC:R-HSA-372790	Signaling by GPCR	1 × 10^−4^	1158	11
DarkGrey	GO:0042989	Sequestering of actin monomers	2 × 10^−3^	12	3
DarkGrey	GO:0032507	Maintenance of protein location in cell	6 × 10^−3^	67	4
DarkGrey	GO:0045185	Maintenance of protein location	2 × 10^−2^	98	4
DarkGrey	GO:0051651	Maintenance of location in cell	3 × 10^−2^	226	5

**Fig. 2:**
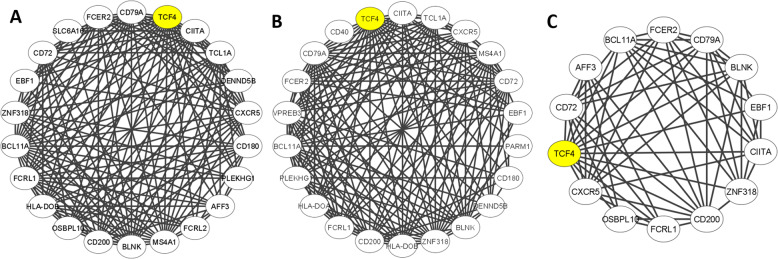
*TCF4* interactome. Network plot of the direct interactor of *TCF4* in the DarkTurquoise module at **A** baseline, **B** 3-year follow-up, and **C** relapse. The figure depicts the connectivity patterns (correlation network adjacencies) between genes in the module.

Among the three semi-conserved modules, only the gene set from the DarkTurquoise module showed significant overlap with gene sets from previous studies (Supplementary Fig. 3). Gene overlap was detected with genes differentially expressed in DLPFC [35] (OR = 2.7, *p* = 0.02) and genes associated with schizophrenia using gene expression imputation across multiple brain regions [36] (OR = 12.8, *p* = 0.01). Among the overlapped genes (*TCF4*, *BCL11A*, *SPRY1*) included in the DarkTurquoise, *TCF4* was present not only in the studies of Fromer et al. [35] and Huckins et al. [36], but also in the GWAS from Pardiñas et al. [37]. Figure 2 shows the network plots of the direct interactors of *TCF4* in the DarkTurquoise module. A loss of density and connectivity could be observed at relapse with respect to baseline, in agreement with the module preservation statistics.

**Fig. 3:**
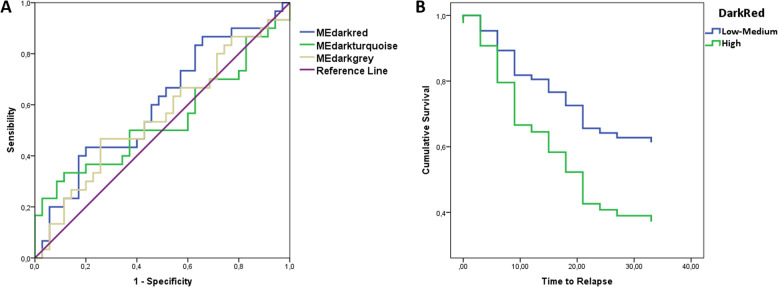
Predictive properties of selected modules. **A** ROC curve analysis of module eigengenes values at baseline of the three semi-conserved modules (DarkRed, DarkGrey, and DarkTurquoise) identified in the present study. **B** Cox regression analysis of the length of time (months from baseline) taken to reach relapse using dichotomized DarkRed baseline module eigengene values.

The predictive properties of semi-conserved modules at baseline were tested using ROC curves (Fig. 3A). To this end, module eigengene values at baseline were used as predictors of relapse. As could be observed, the DarkRed module showed better performances (AUC = 0.603, CI = 0.464-0.742), followed by DarkGrey (AUC = 0.556, CI = 0.414–0.699) and DarkTurquoise (AUC = 0.542, CI = 0.395–0.689), although none of the values achieved significance (*p* > 0.05).

As DarkRed was the module that showed better prediction performance, Kaplan–Meier and Cox regression analysis were used to test its effect on the length of time (months from baseline) taken to reach relapse. To this end, baseline module eigengene values were dichotomized based on percentile distribution, and the highest 75 percentile was chosen as risk variant for the analysis. Patients at the highest 75 percentile (*N* = 20) showed higher risk of suffering a relapse (OR = 2.10, CI = 1.01–4.33, beta = 0.742 ± 0.370, *p* = 0.045), and relapse appeared earlier (21.60 ± 2.74 vs 27.13 ± 1.85 months, *p* = 0.045) than the rest of patients (*N* = 45) (Fig. 3B).

## Discussion

Relapse remains among one of the most challenging aspects of psychosis. In this study, the WGCNA approach was used to identify important genes clustered into modules that are likely enriched for biological pathways that could be dysregulated in relapse. Identified genes will provide new insights into the molecular mechanism involved in relapse and potential biomarkers for relapse prediction.

Using a preservation analysis, our study identified two semi-conserved modules of co-expressed genes (DarkRed and DarkGrey) in both groups after 3 years of follow-up and at relapse. Taking into account that these modules could not be considered state-markers, we hypothesized that genes belonging to these modules could be influenced by antipsychotic treatment and course of disease, and therefore could be related to therapeutic response. Considering that therapeutic response could be one of the predictors of relapse, we tested the relapse predictive properties of these modules at baseline. Patients with higher expression of genes in the DarkRed module showed higher risk of suffering a relapse and earlier appearance of relapse. Interestingly, genes included in the DarkRed module participate in biological processes related to the ubiquitin proteosome system. The ubiquitin proteosome system regulates protein degradation and is a master regulator of neuronal development, influencing neurogenesis, synaptogenesis, and neurotransmission by determining the localization, interaction, and turnover of synaptic proteins [40]. Recently, the ubiquitin proteosome system has been associated with schizophrenia [41] through gene expression studies [42**–**45] and seems to be affected by antipsychotic treatment [46].

Our analysis also identified one module of co-expressed genes that showed evidence of alteration in intramodular connectivity in the relapse samples: the DarkTurquoise module. However, this result could be due to lack of robustness of the modules instead of relapse, as seems to be influenced by module size. It is worth noticing that the genes included in this module, despite being identified in blood samples, showed significant overlap with genes associated with schizophrenia in previous studies analyzing brain gene expression [35, 36]. Among these genes, the member of the helix–loop–helix family transcription factor 4 (*TCF4*)—one of the leading schizophrenia risk genes [47]—stands out [48]. Previous GWAS identified *TCF4* polymorphisms linked with schizophrenia and other psychiatric conditions [37, 49–53] as well as non-neurological genetic diseases [54–56]. Two other genes of the DarkTurquoise module, *BLC11A* and *SPRY1*, overlapped with genes differentially expressed in previous studies analyzing brain gene expression [35, 36]. Interestingly, *BCL11A*, a zinc finger protein that regulates transcription, showed decreased co-expression in multiple schizophrenia cohorts, in both peripheric and central nervous system, using machine learning to predict co-expression at the individual level [57]. Moreover, genetic variants in this gene were found to be associated with schizophrenia [50] and to co-localize with differential DNA methylation sites [58].

Some limitations should be considered in the interpretation of our results. Firstly, the sample size that might also limit the statistical power to detect a difference between groups. Secondly, although the Morinsky–Green scale was used to assess the adherence of the patients, our sample size is not large enough to stratify patients according to it. Therefore, we cannot analyze separately secondary relapse, commonly associated with non-adherence, and natural or primary relapse, which represents relapse in the absence of this influencer [12]. Thirdly, we cannot discard that module preservation changes during the longitudinal analysis are related to age and normal development. To rule out the confounding effect of age in our results a control group would be necessary, formed by healthy volunteers matching the age range of our group with a follow-up of at least 36 months. Lastly, due to the naturalistic design, drug treatment was not controlled; the study participants maintained their usual treatment. Regarding the slightly differences in drug treatment at follow-up, we cannot rule out the possible effect of these differences in the expression of the genes included in semi-preserved modules detected at relapse. Besides these limitations, the strength of this study lies in the inclusion of a consistent well-characterized first-episode schizophrenia patient sample in remission due to its naturalistic and longitudinal design.

To our knowledge this is the first study that compares peripheral gene expression between remission and relapse in a sample of first-episode schizophrenia patients. Although further validation in larger samples is still needed, our results provide new insights into the molecular mechanisms involved in relapse, potential candidates for biomarker discovery, and the development of preventive and therapeutic interventions.

## Supplementary information

Supplementary Material
