# Supplementary material for: A longitudinal study of gene expression in first-episode schizophrenia; exploring relapse mechanisms by co-expression analysis in peripheral blood
Source: Transl Psychiatry. 2021 Oct 19;11:539. doi: 10.1038/s41398-021-01645-8 (PMC8526619; doi:10.1038/s41398-021-01645-8)
Supplement: Supplementary file 1 — Supplementary Material [file 41398_2021_1645_MOESM1_ESM.docx]

**Figure S1**. WGCNA network and module detection. (A) (Sample clustering was conducted to detect outliers. Red color identify the excluded sample (B) Selection of the soft-thresholding powers. The left panel showed the scale-free fit index versus soft-thresholding power. The right panel displayed the mean connectivity versus soft-thresholding power. Power 6 was chose, for which the fit index curve flattens out upon reaching a high value (>0.8) (C) Clustering dendrogram of genes based on a dissimilarity measure (1-TOM), which was then used to group genes into 25 modules in baseline samples. The branches correspond to modules of highly interconnected groups of genes. The height (y-axis) indicates the co-expression distance and the x-axis corresponds to genes. Colors represent the 25 different modules along with gray indicating genes that could not be assigned to any module.


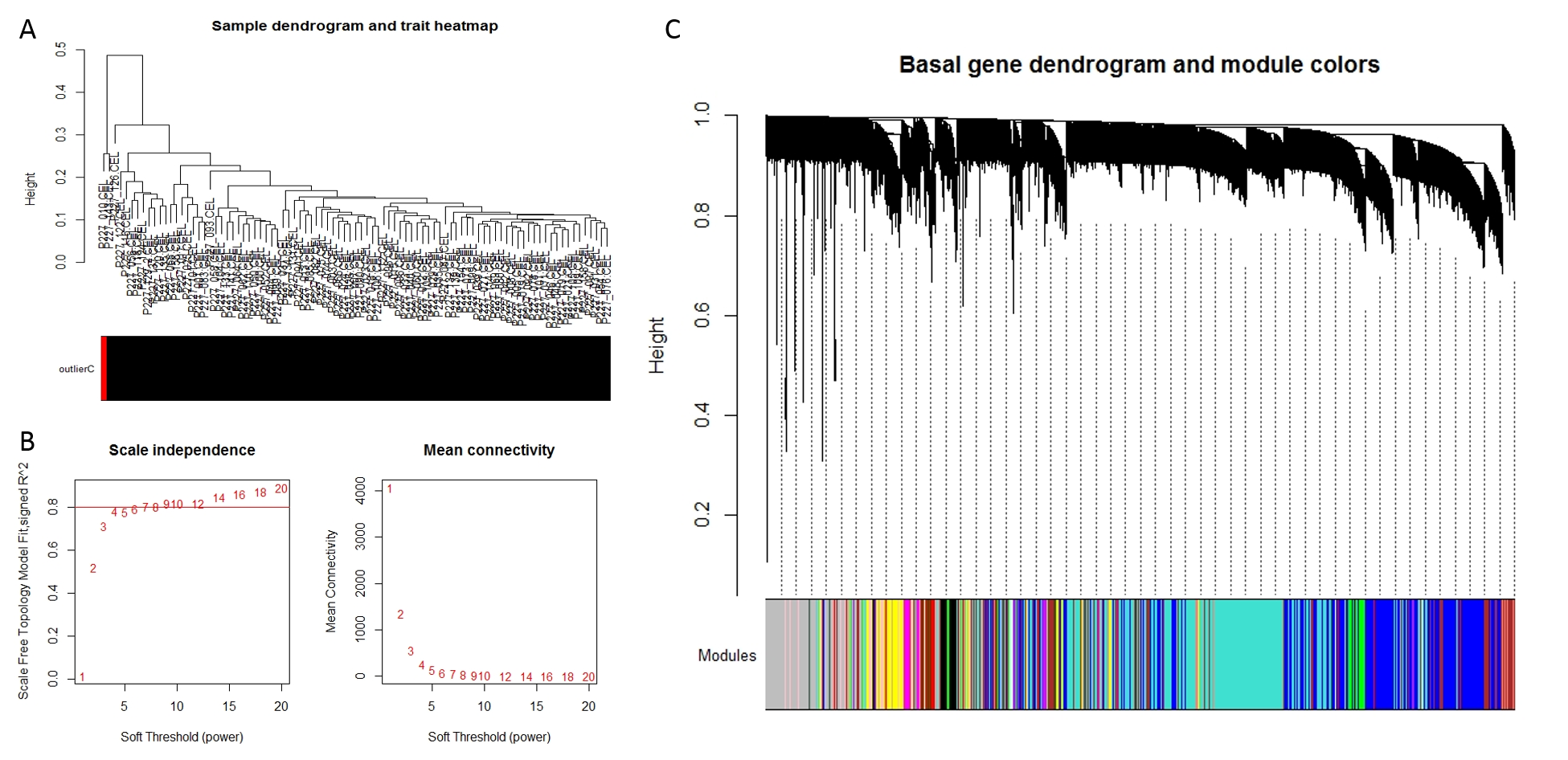


**Figure S2.** Scatter plot of the meadianRank and Zsummary composite module preservation statistic and module size in (A) subset of samples after three-year follow-up, and (B) subset of samples at relapse. The modules with Zsummary > 10 were interpreted as highly preserved, if 2<Zsummary<10 they were defined as semi-conserved and the modules with Zsummary<2 were considered to be non-preserved.

**A.**


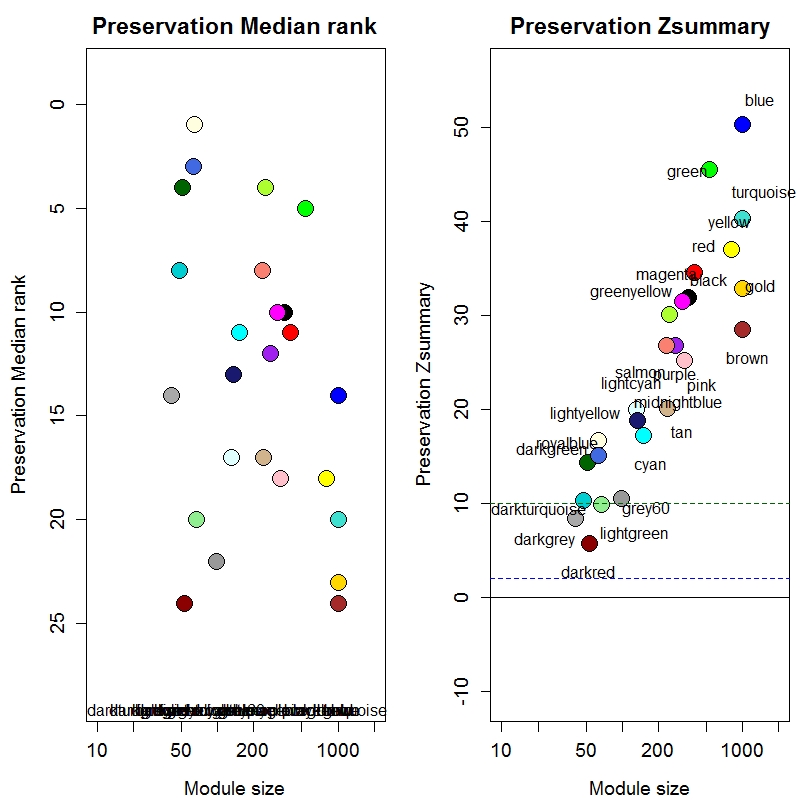


**B.**


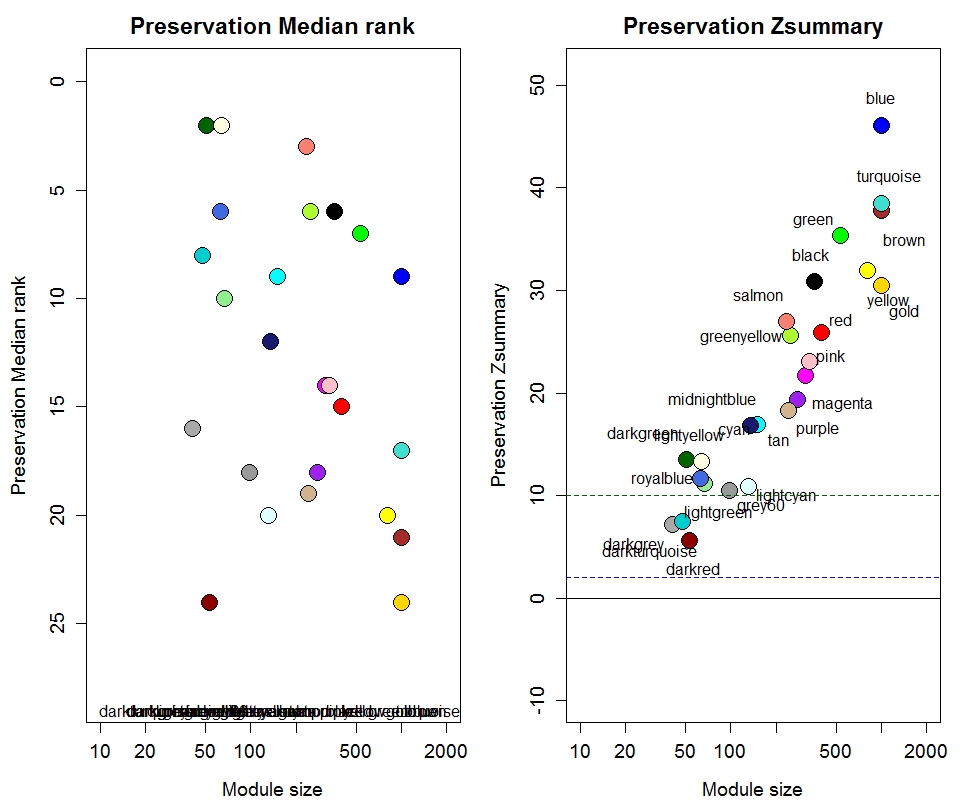


**Figure S3.** Summary statistics (p-value and OR) of the overlap test. Gene sets include: (a) Gandal: differentially expressed genes in dorsolateral prefrontal cortex (DLPFC) (34); (b) Froomer: differentially expressed genes in DLPFC between subjects with schizophrenia (N = 258) versus control (n = 279) subjects (35); (c) Huckins: genes associated with schizophrenia using gene expression imputation (Transcriptome Wide Analysis, TWAS) across multiple brain regions in 40,299 schizophrenia cases and 65,264 matched controls (36); (d) PGRS_PGC: genes that have association with common genome wide association study (GWAS) meta-analysis of the CLOZUK and independent Psychiatric Genomic Consortium (PGC) datasets, excluding related and overlapping samples (total of 40,675 cases and 64,643 controls) (37).

**
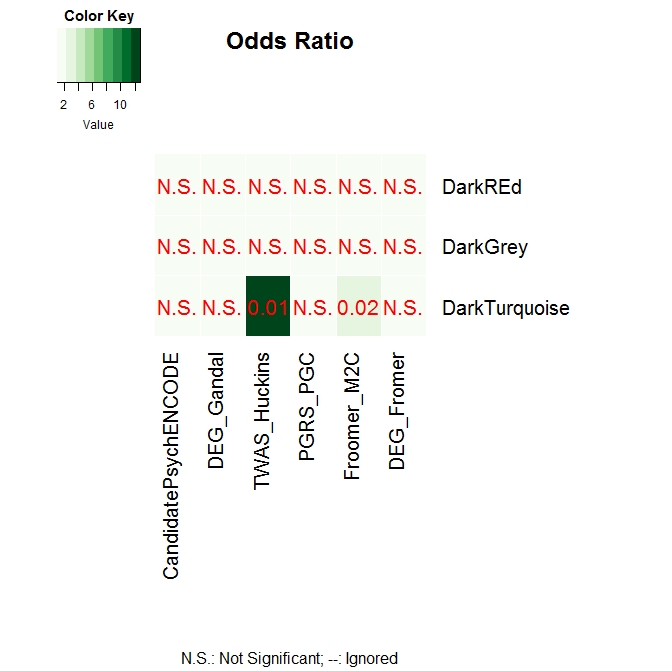
**

**Table S1.** Genes included in the three semi-preserved modules identified in the present study.

| DarkREd | DarkGrey | DarkTurquoise |
| --- | --- | --- |
| CYP4Z1 | CMPK1 | FAM177B |
| PALMD | RPL22 | FCRL5 |
| OR10J3 | TAF12 | FCRL2 |
| C2orf78 | SELENOF | FCRL1 |
| POTEE | LAMTOR5 | BCL11A |
| ERICH2 | POLR3GL | AFF3 |
| POTEI | ACP1 | KCNH8 |
| OR5H1 | TMSB10 | ZNF860 |
| MAATS1 | SF3B6 | OSBPL10 |
| LRRC15 | BRK1 | CD200 |
| USP17L10 | H2AZ1 | PARM1 |
| USP17L13 | CAMLG | SPRY1 |
| USP17L15 | SKP1 | EBF1 |
| USP17L17 | ABRACL | CD180 |
| USP17L19 | MPC1 | COL19A1 |
| PSAPL1 | TMEM14B | PLEKHG1 |
| OR2Y1 | PSMB8-AS1 | HLA-DOA |
| SCGN | RPS10-NUDT3 | BACH2 |
| TCP11 | SDHAF3 | HLA-DOB |
| ZPBP | TMEM60 | ZNF318 |
| USP17L1 | MED30 | TSPAN13 |
| USP17L4 | TMSB4X | BLK |
| ZNF705G | NHS | PNOC |
| USP17L7 | BEX4 | PLPP5 |
| IFNA4 | TMSB4Y | CD72 |
| FAM205C | GSTO1 | PAX5 |
| TEX48 | HTATIP2 | BLNK |
| SSX6P | SDHD | LARGE2 |
| CXorf66 | ERP29 | MS4A1 |
| C10orf126 | DYNLL1 | CXCR5 |
| OR51T1 | GPN3 | BCL7A |
| OR52N2 | NDUFA12 | BHLHE41 |
| MRGPRX3 | PSMB5 | DENND5B |
| OR4C12 | DUT | TCL1A |
| OR6C74 | EID1 | FAM30A |
| OR6C1 | SRP14 | CIITA |
| PCOTH | RPAP1 | CD79B |
| SLITRK6 | SELENOS | P2RX5 |
| OR11H12 | SDF2 | P2RX5-TAX1BP3 |
| OR4N2 | SUMO2 | KLHL14 |
| OR11H2 | UQCRFS1 | TCF4 |
| GOLGA6A |  | NIBAN3 |
| TP53TG3D |  | CD79A |
| EMC8 |  | FCER2 |
| CNTNAP4 |  | SLC6A16 |
| KRTAP1-5 |  | PKIG |
| POTEC |  | CD40 |
| OR7D2 |  | VPREB3 |
| PRG1 |  |  |
| DNASE2 |  |  |
| ZNF442 |  |  |
| KRTAP19-7 |  |  |
| OR11H1 |  |  |
